# Convergent Evolution and the Diverse Ontogenetic Origins of Tendrils in Angiosperms

**DOI:** 10.3389/fpls.2018.00403

**Published:** 2018-04-03

**Authors:** Mariane S. Sousa-Baena, Neelima R. Sinha, José Hernandes-Lopes, Lúcia G. Lohmann

**Affiliations:** ^1^Laboratório de Sistemática, Evolução e Biogeografia de Plantas Vasculares, Departamento de Botânica, Instituto de Biociências, Universidade de São Paulo, São Paulo, Brazil; ^2^Department of Plant Biology, University of California, Davis, Davis, CA, United States; ^3^Genomics and Transposable Elements Laboratory (GaTE-Lab), Departamento de Botânica, Instituto de Biociências, Universidade de São Paulo, São Paulo, Brazil

**Keywords:** climbing habit, helical growth, lianas, ontogenetic origin, primary homology, recurrent evolution, tendrils, vines

## Abstract

Climbers are abundant in tropical forests, where they constitute a major functional plant type. The acquisition of the climbing habit in angiosperms constitutes a key innovation. Successful speciation in climbers is correlated with the development of specialized climbing strategies such as tendrils, i.e., filiform organs with the ability to twine around other structures through helical growth. Tendrils are derived from a variety of morphological structures, e.g., stems, leaves, and inflorescences, and are found in various plant families. In fact, tendrils are distributed throughout the angiosperm phylogeny, from magnoliids to asterids II, making these structures a great model to study convergent evolution. In this study, we performed a thorough survey of tendrils within angiosperms, focusing on their origin and development. We identified 17 tendril types and analyzed their distribution through the angiosperm phylogeny. Some interesting patterns emerged. For instance, tendrils derived from reproductive structures are exclusively found in the Core Eudicots, except from one monocot species. Fabales and Asterales are the orders with the highest numbers of tendrilling strategies. Tendrils derived from modified leaflets are particularly common among asterids, occurring in Polemoniaceae, Bignoniaceae, and Asteraceae. Although angiosperms have a large number of tendrilled representatives, little is known about their origin and development. This work points out research gaps that should help guide future research on the biology of tendrilled species. Additional research on climbers is particularly important given their increasing abundance resulting from environmental disturbance in the tropics.

## Introduction

Biologists have long investigated the reasons why some lineages are more diverse than others (Magallón and Sanderson, 2001). Highly diverse clades have often been associated with key innovations, i.e., the possession of novel morphological features that allowed those lineages to exploit previously unused or under-utilized resources, leading to increased diversification rates (Cracraft, 1990). Many traits are thought to be key innovations, however relatively few have actually been tested using a phylogenetic approach, and well-documented key-innovations are still scarce in the literature. The acquisition of climbing habit in flowering plants constitutes a great key innovation example, with climbing clades being substantially more diverse than their non-climbing sister taxa (Gianoli, 2004).

Climbers are abundant in tropical forests, where they constitute a major functional plant type (Schnitzer, 2005). Due to the weedy nature of some species, they can become serious invaders. For instance, *Pueraria montana*, a species native from Asia known as Kudzu, currently occupies over 3 million hectares of the eastern United States, causing serious economic and environmental problems (Blaustein, 2001; Forseth and Innis, 2004). Within forests, the distribution of climbing mechanisms varies spatially. For instance, tendril climbers are more prominent in early successional environments, forest edges, and disturbed sites characterized by thinner host stem diameters (DeWalt et al., 2000), which might be correlated to the increased abundance and density of climbing plants after forest disturbance (Gallagher and Leishman, 2012). Owing to these factors, research on ecological, evolutionary and taxonomic aspects of climbing plants increased substantially over the past years (Gallagher and Leishman, 2012).

The climbing habit evolved independently in angiosperms, gymnosperms, and ferns (Isnard and Feild, 2015). Climbing representatives are found in nine families of pteridophytes and two families of gymnosperms (Gianoli, 2015), whereas the climbing habit evolved in nearly all lineages of angiosperms, being found from basal angiosperms (e.g., Austrobaileyales) to asterids (e.g., Asteraceae and Bignoniaceae; Isnard and Feild, 2015). Approximately 65% of eudicot and magnoliid orders (Gentry, 1991), as well as 160 angiosperm families include at least one climbing species (Gianoli, 2015). Angiosperms have evolved various strategies for climbing, such as twining stems (the most common climbing mechanism in angiosperms), tendrils, hooks, and sticky adventitious roots (Vaughn and Bowling, 2010; Burnham and Revilla-Minaya, 2011). As such, the climbing habit is associated with various structural modifications on stems (Angyalossy et al., 2014), leaves (Hofer et al., 2009; Sousa-Baena et al., 2014a,b), and roots (Groot et al., 2003). A few families exhibit more than one climbing strategy (Burnham and Revilla-Minaya, 2011), suggesting that climbing mechanism might be related to phylogeny (i.e., with closely-related species resembling each other more than expected by chance; Blomberg and Garland, 2002). Highly diverse families of climbing plants usually have additional specific climbing mechanisms besides stem twining, indicating that successful speciation might be correlated with the development of more specialized climbing strategies such as tendrils (Gentry, 1991).

Tendrils are specialized organs with filiform shape that have the ability to twine around other structures through helical growth (Darwin, 1875). In vines, the growing climbing organ, tendril or stem, finds suitable support through circumnutation, a bending helical movement of growing organs. The twining process initiates within seconds or minutes after the touch-sensitive region of tendrils located near their tips contacts suitable support (Jaffe and Galston, 1968). The organ bends to that side while older plant parts initiate revolutions caused by asymmetrical growth pulses of the entire young stem. In other words, the tip draws an ellipse and the organ rotates around a central axis during growth (Kiss, 2006; Migliaccio et al., 2013). Organ bending is caused by water imbalance on one side of the organ, which increases cell turgidity, causing them to elongate (Migliaccio et al., 2013). During the helical growth and coiling movements, tendrils display an increase in respiration rate (Riehl and Jaffe, 1982), as well as changes in membrane permeability associated with an increase in solute efflux (Jaffe and Galston, 1968; Jaffe, 1975; Liß and Weiler, 1994). All of these processes are coordinated by complex hormonal regulation pathways (Jaffe, 1975).

Tendrils evolved multiple times during the history of angiosperms, representing a beautiful case of convergent evolution. They are found in magnoliids (e.g., Annonaceae), monocots (e.g., Colchicaceae and Smilacaceae), early eudicots (Papaveraceae and Ranunculaceae), rosids (e.g., Cucurbitaceae, Fabaceae, and Passifloraceae), and asterids (e.g., Apocynaceae, Asteraceae, and Bignoniaceae). In fact, the genetic capacity to grow as a tendrilled climbing plant existed in some of the earliest land plants (Vaughn and Bowling, 2010). Indeed, in the climbing Permian pteridosperms, *Lescuropteris genuina*, had tendrils derived from terminal leaflets, while *Blanzyopteris praedentata* had lateral branches modified into tendrils with ramifications terminating in adhesive pads (Krings et al., 2003). In pteridophytes, tendrilled species are found, for instance, in the genus *Lygodium*, whose species bear leaves in which the rachis acquires helical growth that functions as a tendril.

Generally simple in structure, tendrils undergo complex physiological changes during development that result from processes such as circumnutation and contact coiling (Darwin, 1875; Jaffe and Galston, 1968; Jaffe et al., 2002). Different hypotheses have been proposed to explain circumnutation. Some of the earliest studies considered circumnutation as an autonomous process (i.e., originating from internal plant factors, independent of the influence of external forces) (Darwin, 1875). Other studies hypothesized that circumnutation is induced by gravitropism and an inner oscillator (Brown, 1993), two independently operating mechanisms (Migliaccio et al., 2013). Recent studies suggested that phototropism, thigmotropism, gravitropism, microfibril orientation in xylem, microtubule orientation in cells, epidermal cell anisotropism, and anatomical asymmetry might also be associated with circumnutation (Burnham and Revilla-Minaya, 2011). While various hypotheses about circumnutation are available, a full understanding of their structural mechanism and underlying molecular controls remains unknown (Bowling and Vaughn, 2009; Gerbode et al., 2012).

Little is known about the development, origin and molecular regulation of tendrils outside *Pisum* (peas, Vitaceae), *Cucumis* (cucumber, Cucurbitaceae), and *Vitis* (grapevine, Vitaceae). Studies on the underlying molecular basis of tendril development suggest their molecular regulation is complex as even tendrils with the same ontogenetic origin are controlled by diverse gene networks (Sousa-Baena et al., 2018). Specifically, helical growth and coiling in tendrils seems to be generated by gelatinous fibers via asymmetric contraction of the fiber ribbon owing to differential desiccation (Bowling and Vaughn, 2009; Gerbode et al., 2012). However, a possible link between changes in cytoskeletal elements and helical growth in natural climbing plants has been recently established (Smyth, 2015; Sousa-Baena et al., 2018). The ability to perform helical growth is found in ferns, gymnosperms, basal angiosperms, magnoliids, monocots, and eudicots, indicating that helical growth might actually represent a case of deep homology. As such, studies focusing on genes controlling cytoskeletal components during tendril development may shed light on mechanisms of convergent evolution enabled by deep homology.

Here, we examine the convergent evolution of tendrils within angiosperms, focusing on their ontogenetic origin and development. We adopt a broad definition of tendril: any organ that undergoes helical growth, performing the role of climbing (exclusively or not), except from the twining shoots found in stem twiners. We also review the development and structure of tendrils with terminal adhesive pads, a special tendril type.

## Straight to the top: distantly related taxa use ontogenetically distinct but functionally similar structures to climb

**Figure 1:**
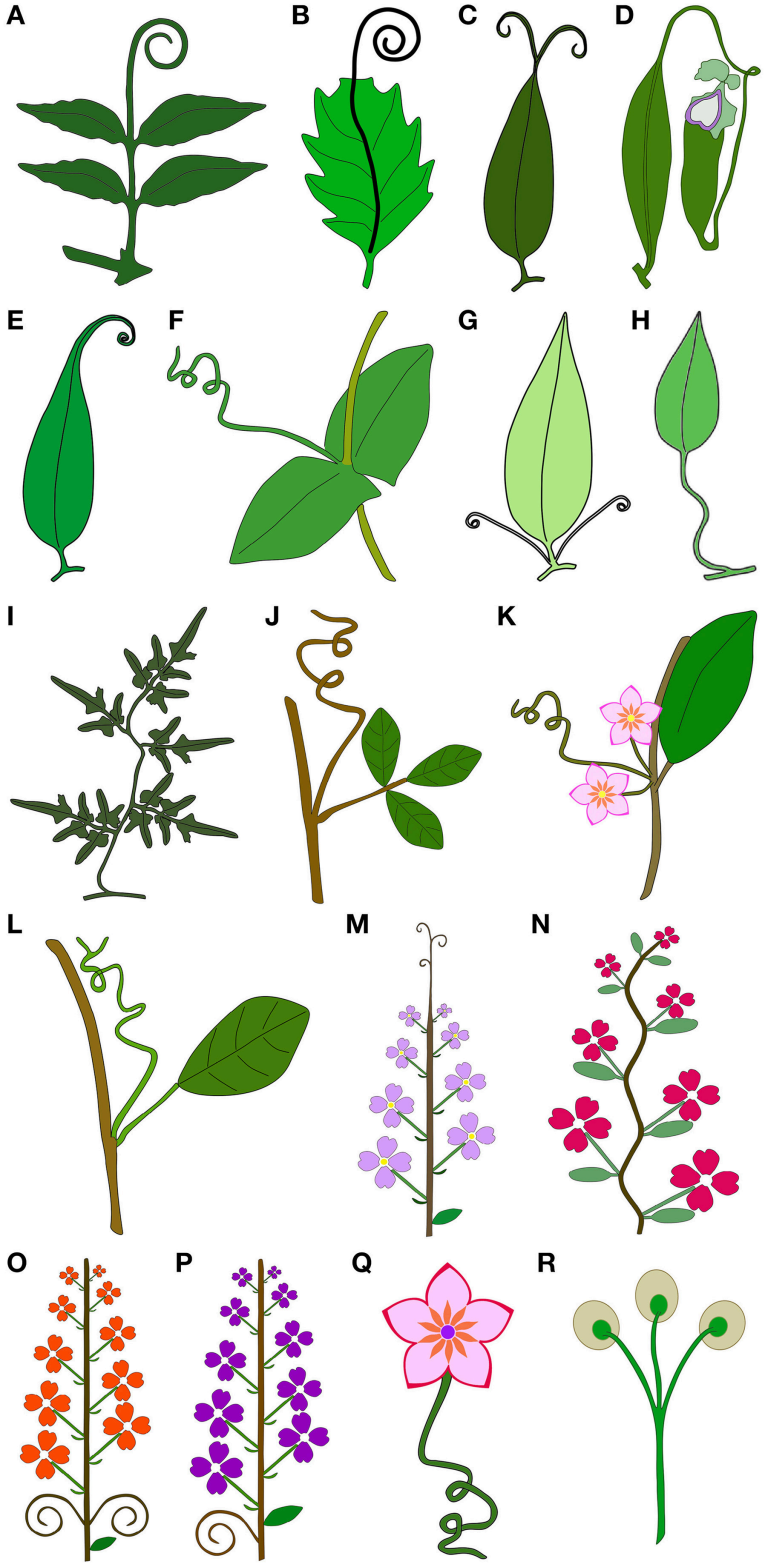
Schematic drawings illustrating the 17 tendril types classified in this work, and tendrils terminating in apical adhesive pads. **(A–J)** Tendrils derived from vegetative organs. **(A)** Tendrils derived from modified terminal leaflets. **(B)** Tendril derived from prolonged midrib. **(C)** Tendrils derived from prolonged bifurcated tips of the leaf midribs. **(D)** Tendrils derived from the modified petioles, representing a transitory structure that acquires functions other than climbing in later stages of leaf development. **(E)** Tendrils derived from a modified leaf tip. **(F)** Whole leaf modified into a simple tendril. **(G)** Tendrils derived from petiole duplication. **(H)** Tendrils derived from petioles that acquire the capacity for helical growth. **(I)** Tendrils derived from modified compound leaf rachis that acquires the capacity for helical growth, becoming voluble. **(J)** Tendril derived from a modified shoot. **(K–Q)** Tendrils derived from reproductive organs. **(K)** Tendrils derived from the tip of the reduced inflorescence apex. **(L)** Tendril derived from modified inflorescences. **(M)** Tendrils derived from modified inflorescence apices. **(N)** Tendrils derived from modified inflorescence rachis that acquires the capacity for helical growth, becoming voluble. **(O)** Tendrils derived from inflorescence lateral branches. **(P)** Tendrils derived from inflorescence peduncles. **(Q)** Tendril derived from flower pedicels that acquire the capacity for helical growth. **(R)** Tendrils terminating in apical adhesive pads.

**Figure 2:**
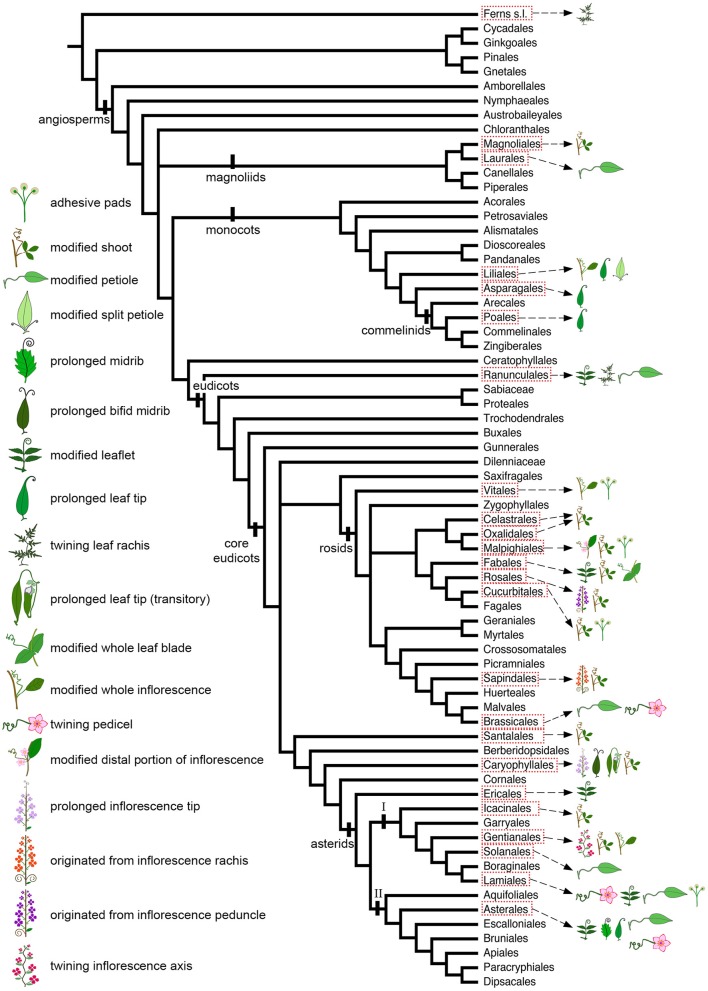
Angiosperm phylogeny modified from Stevens (2001 onwards) to include Icacinales (following the APG IV; The Angiosperm Phylogeny Group, 2016), illustrating the distribution of tendrils with different ontogenetic origins, as well as the presence of adhesive pads, across angiosperm orders.

**Figure 3:**
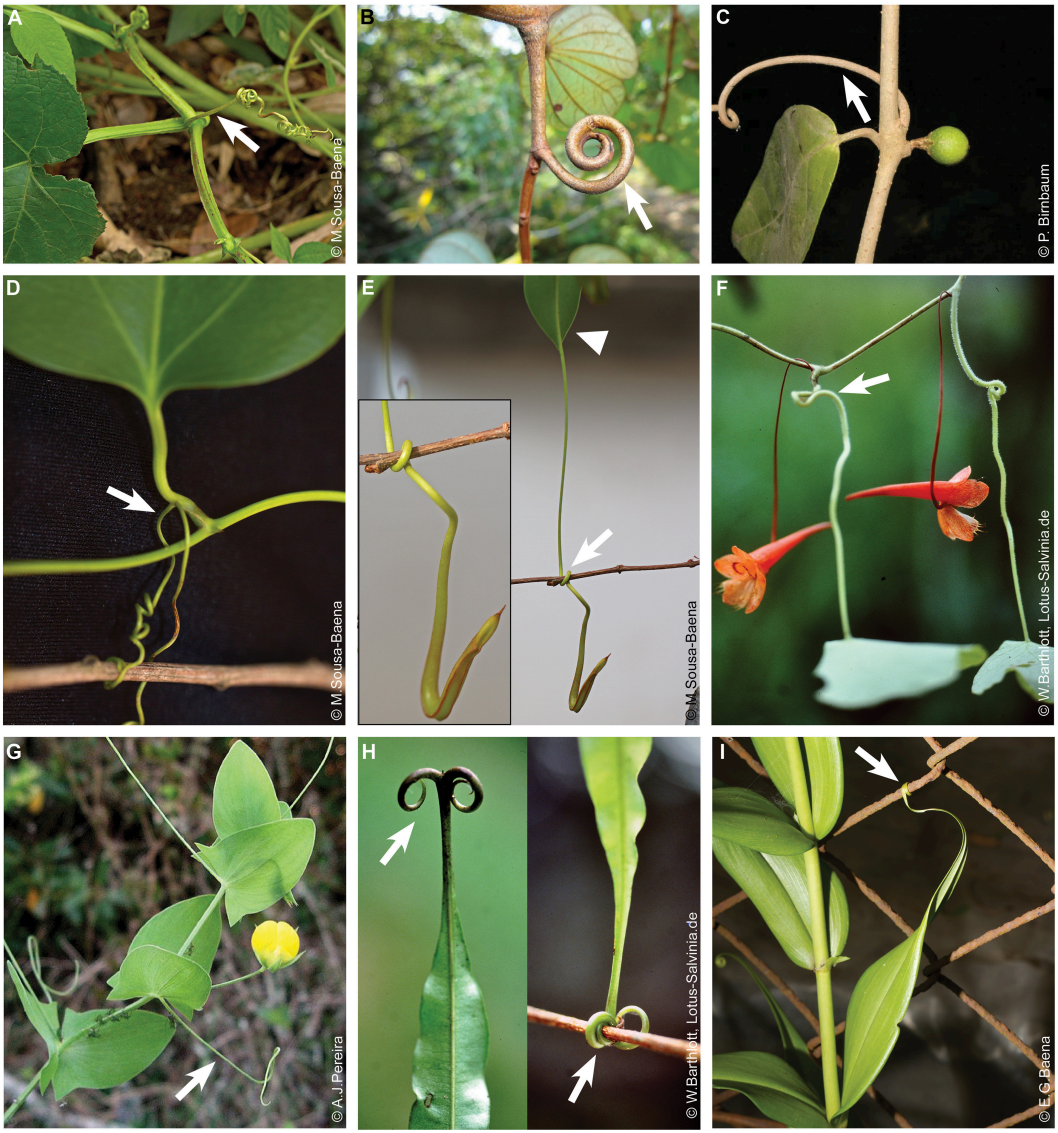
Tendrils derived from vegetative structures. **(A–C)** Tendrils derived from shoots. **(A)** *Cucurbita pepo* (Cucurbitaceae). **(B)** *Bauhinia semibifida* (Fabaceae). Image source: www.NatureLoveYou.sg. **(C)** *Landolphia dulcis* (Apocynaceae). **(D–I)** Tendrils derived from leaves or leaf parts. **(D)** *Smilax* sp. (Smilacaceae). Tendrils derived from the petiole. **(E)** *Nepenthes* sp. (Nepenthaceae). Detail of tendril and developing pitcher on the left. Arrowhead indicates the leaf blade from which a tendril is emerging. **(F)** Tendrils derived from petioles in *Tropaeolum repandum* (Tropaeolaceae). This image was reproduced with permission from [Wilhelm Barthlott]. **(G)** Whole leaf blade transformed into a tendril in *Lathyrus aphaca* (Fabaceae). Image source: www.flora-on.pt. **(H)** *Triphyophyllum peltatum* (Dioncophyllaceae). On the left side a detail illustrating the tendril grasping a branch. Images were reproduced with permission from [Wilhelm Barthlott]. **(I)** Tendrils originated from the leaf tip in *Gloriosa superba* (Colchicaceae). Arrows indicate tendrils. Image source of pictures in **(F,H)** (https://www.flickr.com/photos/lotus-salvinia).

**Figure 4:**
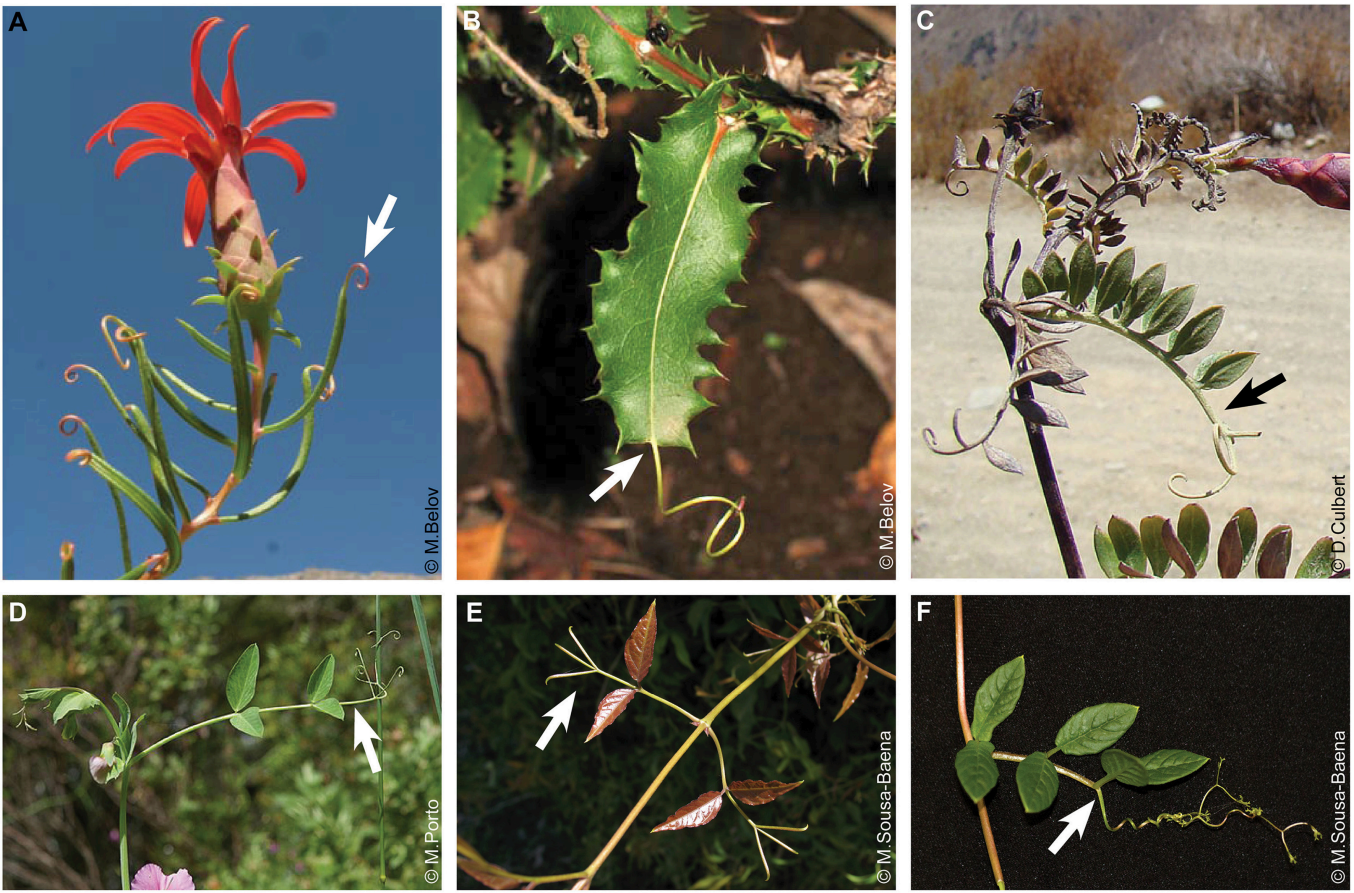
Tendrils derived from leaf parts. **(A)** Tendrils derived from the leaf tip in *Mutisia subulata* (Asteraceae). **(B)** Tendril derived from a prolonged leaf midrib in *Mutisia brachyanta*. Images in **(A,B)** were reproduced with permission from [Michail Belov] (Image source: http://www.chileflora.com). **(C-F)** Tendrils derived from modified terminal leaflets. **(C)** *Mutisia acuminata*. **(D)** *Pisum sativum* (Fabaceae). Image source: www.flora-on.pt. **(E)** *Dolichandra unguis-cati* (Bignoniaceae). **(F)** *Cobaea scandens* (Polemoniaceae). Arrows indicate tendrils.

Tendrils are a key feature associated with the evolution of the climbing habit (Gentry, 1980). We have identified 17 tendril types based on their ontogenetic origin and growth pattern (Figure 1), with most tendrils types evolving more than once within angiosperms (Figure 2). Tendrils are found in many angiosperm clades, occurring in magnoliids (Laurales), early eudicots (Ranunculales, Ranunculaceae; Tamura, 1993), rosids (Fabales, Fabaceae; Darwin, 1875), asterids I (Lamiales, Bignoniaceae; Lohmann and Taylor, 2014), and asterids II (Asterales, Asteraceae; Hind, 2007), among others (Figure 2). Among the neotropical climbing families that bear tendrils, Bignoniaceae, Cucurbitaceae, Fabaceae, Loganiaceae, Passifloraceae, Sapindaceae, and Vitaceae are the most species-rich groups (Putz, 1984). While some tendril types seem to be more common within particular lineages, other clades seem to be highly diverse in terms of climbing strategies. The orders with larger numbers of tendrilling strategies are Fabales (rosid I) and Asterales (asterid II) (Table 1). In the former, Fabaceae and Polygalaceae evolved tendrils, comprising three different strategies: (i) whole leaves modified into tendrils (found in the Fabaceae exclusively (Figure 3G); (ii) terminal leaflets modified into tendrils (found in the Fabaceae exclusively; Figure 4D); and (iii) shoots modified into tendrils (found in the Fabaceae and Polygalaceae; Figure 3B) (Table 1). In Asterales, tendrils evolved in Asteraceae and Campanulaceae, comprising five different strategies: (i) simple leaves with tendrils derived from the leaf tip. The leaf blade becomes gradually narrower toward the tip, which is prolonged and acquires helical growth (found in *Mutisia*, Asteraceae; Figure 4A), (ii) simple leaves with tendrils derived from prolonged leaf midribs. The leaf blade ends abruptly, forming a truncate leaf apex from which a tendril derived from the midrib arises (found in *Mutisia*, Asteraceae; Figure 4B), (iii) compound leaves with tendrils derived from terminal leaflets (found in *Mutisia*, Asteraceae; Figure 4C), (iv) twining pedicels (found in *Canarina*, Campanulaceae), and, (v) twining petioles (found in *Canarina*, Campanulaceae) (Table 1).

**Table 1:** Tendril bearing angiosperm families, followed by the tendril ontogenetic origins, and reference to the literature.

**Order**	**Family**	**Genus**	**Tendril origin**	**Reference**
Magnoliales	Annonaceae (1)	*Uvaria*	Modified stem	Johnson, 2003
		*Monanthotaxis*	Modified stem	Hegarty and Caballé, 1991
Laurales	Hernandiaceae (1)	*Illigera*	Twining petiole	Kubitzki, 1993
Liliales	Smilacaceae (1)	*Smilax*	“Dédoublement of the petiole”/modified stipule	Arber, 1920
	Liliaceae (1)	*Fritillaria*	Prolonged leaf tip	Arber, 1920
	Colchicaceae (1)	*Gloriosa*	Prolonged leaf tip	Nordenstam, 1998
		*Littonia*	Prolonged leaf tip	Nordenstam, 1998
	Petermanniaceae (1)	*Petermannia*	Modified whole inflorescence	Tomlinson and Ayensu, 1969
Asparagales	Asparagaceae (1)	*Polygonatum*	Prolonged leaf tip	Arber, 1920
Poales	Flagellariaceae (1)	*Flagellaria*	Prolonged leaf tip /thickened prolonged leaf midrib	Takhtajan, 1997; Appel and Bayer, 1998; Hesse et al., 2016
Ranunculales	Papaveraceae (2)	*Dicentra*	Modified terminal leaflet	Kadereit, 1993
		*Disocapnos*	Modified terminal leaflet	Kadereit, 1993
		*Trigonocapnos*	Modified terminal leaflet	Kadereit, 1993
		*Ceratocapnos*	Modified terminal leaflet	Kadereit, 1993
		*Cysticapnos*	Modified terminal leaflet, voluble rachis and petiolule	Hidalgo et al., 2012
		*Adlumia*	Modified terminal leaflet	Gentry, 1991
	Ranunculaceae (2)	*Clematis*	Twining petiole and/or twining rachis	Tamura, 1993
		*Naravelia*	Modified terminal leaflet	Tamura, 1993
	Menispermaceae (1)	*Cissampelos*	Twining petiole	Stevens (2001 onwards)
Vitales	Vitaceae (1)	*Vitis*	Modified inflorescence/modified extra-axillary branch	Tucker and Hoefert, 1968; Shah and Dave, 1970; Boss and Thomas, 2002; Wen, 2007; Zhang et al., 2015; Gerrath et al., 2017
		*Ampelocissus*	Modified inflorescence	Gerrath et al., 2017
		*Parthenocissus*	Modified inflorescence/modified extra-axillary branch, adhesive pads +/−	Shah and Dave, 1970; Wen, 2007; Gerrath et al., 2017
		*Tetrastigma*	Modified inflorescence /modified extra-axillary branch, adhesive pads +/−	Shah and Dave, 1970; Gerrath et al., 2017
		*Cyphostemma*	Modified inflorescence	Gerrath et al., 2017
		*Cissus*	Modified inflorescence, adhesive pads +/−	Gerrath and Posluszny, 1994; Wen, 2007; Gerrath et al., 2017
		*Ampelopsis*	Modified inflorescence /modified extra-axillary branch	Shah and Dave, 1970; Gerrath and Posluszny, 1989; Gerrath et al., 2017
		*Pterisanthes*	Modified inflorescence	Ickert-Bond et al., 2015
		*Rhoicissus*	Modified inflorescence	Gerrath et al., 2004, 2017
		*Nekemias*	Modified inflorescence	Gerrath et al., 2017
Celastrales	Celastraceae (1)	*Anthodon*	Modified stem	Burnham and Revilla-Minaya, 2011; Zulqarnain, 2014
Oxalidales	Connaraceae (1)	*Rourea*	Modified stem	Acevedo-Rodríguez, 2005; Burnham and Revilla-Minaya, 2011
Malpighiales	Passifloraceae (1)	*Passiflora*	Modified terminal flower /modified first-order axis of inflorescence /central flower pedicel /modified stem, adhesive pads +/−	Shah and Dave, 1971a; Krosnick and Freudenstein, 2005; Feuillet and MacDougal, 2006; Bohn et al., 2015
		*Paropsia*	Modified first-order axis of inflorescence	Krosnick and Freudenstein, 2005
		*Hollrungia*	Modified first-order axis of inflorescence	Krosnick and Freudenstein, 2005
		*Adenia*	Modified first-order axis of inflorescence	Krosnick and Freudenstein, 2005
	Lophopyxidaceae (1)	*Lophopyxis*	Modified stem	Stevens, 2001 (onwards); Takhtajan, 1997
Fabales	Fabaceae (3)	*Pisum*	Modified terminal leaflet /determined rachis	Darwin, 1875; Tattersall et al., 2005
		*Vicia*	Modified terminal leaflet/determined rachis	Darwin, 1875; Tattersall et al., 2005
		*Lathyrus*	Modified terminal leaflet/determined rachis	Darwin, 1875; Tattersall et al., 2005
			Whole leaf modified into tendril	Darwin, 1875
		*Entada*	Modified terminal leaflet/determined rachis	Gentry, 1991
		*Bauhinia*	Modified stem	Fisher and Blanco, 2014
	Polygalaceae (1)	*Securidaca*	Modified stem	Acevedo-Rodríguez, 2005
Rosales	Rhamnaceae (3)	*Gouania*	Originated from the inflorescence peduncle	Medan and Schirarend, 2004
			Modified stems, and inflorescence branches	Cremers, 1974; Tortosa, 2005
		*Johnstonia*	Modified stems	Tortosa, 2005
		*Reissekia*	Modified stems	Tortosa, 2005
		*Alvimiantha*	Modified stems	Tortosa, 2005
		*Helinus*	Derived from the inflorescence peduncle	Medan and Schirarend, 2004
Cucurbitales	Cucurbitaceae (2)	*Cucurbita*	Ramification of the axillar bud/modified stem	Sensarma, 1955; Schaefer and Renner, 2011
		*Cucumis*	Ramification of the axillar bud/modified stem	Sensarma, 1955; Schaefer and Renner, 2011
		*Citrullus*	Ramification of the axillar bud/modified stem	Sensarma, 1955; Schaefer and Renner, 2011
		*Coccinia*	Ramification of the axillar bud/modified stem	Sensarma, 1955; Schaefer and Renner, 2011
		*Lagenaria*	Ramification of the axillar bud/modified stem	Sensarma, 1955; Schaefer and Renner, 2011
		*Momordica*	Ramification of the axillar bud/modified stem	Sensarma, 1955; Schaefer and Renner, 2011
		*Mukia*	Ramification of the axillar bud/modified stem	Sensarma, 1955; Schaefer and Renner, 2011
		*Thladiantha*	Derived from a stem-stipule complex	Sensarma, 1955
		*Lupha*	Derived from a stem-stipule complex	Sensarma, 1955
		*Echinocyctis*	Modified stem	Gerrath et al., 2008
		*Alsomitra*	Modified stem, adhesive pads +/−	Kocyan et al., 2007
		*Trichosanthes*	Modified stem, adhesive pads +/−	Kocyan et al., 2007
		*Polyclathra*	Modified stem, adhesive pads +/−	Kocyan et al., 2007
		*Neoalsomitra*	Modified stem, adhesive pads +/−	Kocyan et al., 2007
		*Bayabusua*	Modified stem, adhesive pads +/−	Kocyan et al., 2007
Sapindales	Sapindaceae (2)	*Lophostigma*	Tendril pair at the inflorescence rachis base	Acevedo-Rodríguez, 2005
		*Serjania*	Modified stem	Acevedo-Rodríguez, 2005
		*Cardiospermum*	Tendril pair at the inflorescence rachis base	Acevedo-Rodríguez, 2005
		*Thinouia*	Tendril pair at the inflorescence rachis base	Villagra and Romaniuc Neto, 2011
		*Paullinia*	Tendril pair at the inflorescence rachis base	Acevedo-Rodríguez, 2005
Brassicales	Tropaeolaceae (2)	*Tropaeolum*	Twining petioles and pedicels	Bayer and Appel, 2003
Santalales	Olacaceae (1)	*Erythropalum*	Modified stem	Malécot and Nickrent, 2008
Caryophyllales	Polygonaceae (2)	*Brunnichia*	Terminal portion of the inflorescence peduncle/terminal portion of the inflorescence axis	Brandbyge, 1993; Burke and Sanchez, 2011
		*Antigonon*	Terminal portion of the inflorescence peduncle/terminal portion of the inflorescence axis	Shah and Dave, 1971b; Brandbyge, 1993; Burke and Sanchez, 2011
		*Afrobrunnichia*	Modified stem	Stevens (2001 onwards); Cremers, 1974
	Nepenthaceae (1)	*Nepenthes*	Modified leaf tip	Gentry, 1991
	Dioncophyllaceae (1)	*Triphyophyllum*	Prolonged bifurcated tip of the leaf midrib	Takhtajan, 1997; Chase et al., 2009
		*Dioncophyllum*	Prolonged bifurcated tip of the leaf midrib	Porembski and Barthlott, 2003
		*Habropetalum*	Prolonged bifurcated tip of the leaf midrib	Porembski and Barthlott, 2003
Ericales	Polemoniaceae (1)	*Cobaea*	Modified terminal leaflet	Wilken, 2004
Icacinales	Icacinaceae (2)	*Iodes*	Modified inflorescence/non-axillary branch tendrils	Cremers, 1974; Gentry, 1991
		*Polyporandra*	Extra-axillary or intrapetiolar tendrils	Stevens (2001 onwards)
Gentianales	Loganiaceae (1)	*Strychnos*	Modified stem	Cremers, 1973
	Apocynaceae (3)	*Pacouria*	Twining inflorescence axis	Gentry, 1991; Simões et al., 2007
		*Landolphia*	Modified stem	Persoon et al., 1992
		*Willughbeia*	Modified whole inflorescence	Middleton, 1993
Solanales	Solanaceae (1)	*Solanum*	Twining petiole	Knapp, 2013
Lamiales	Bignoniaceae (2)	*Perianthomega*	Twining petiole and rachis	Lohmann, 2003
		*Adenocalymma*	Modified terminal leaflet	Lohmann, 2003
		*Pyrostegia*	Modified terminal leaflet	Lohmann, 2003
		*Mansoa*	Modified terminal leaflet, adhesive pads +/−	Lohmann and Taylor, 2014; Sousa-Baena et al., 2014b
		*Amphilophium*	Modified terminal leaflet, adhesive pads +	Lohmann and Taylor, 2014; Sousa-Baena et al., 2014a,b
		*Bignonia*	Modified terminal leaflet, adhesive pads +/−	Darwin, 1875; Lohmann and Taylor, 2014; Sousa-Baena et al., 2014a,b
		*Manaosella*	Modified terminal leaflet, adhesive pads +	Lohmann and Taylor, 2014
		*Dolichandra*	Modified terminal leaflet	Sousa-Baena et al., 2014a,b
		*Fridericia*	Modified terminal leaflet	Sousa-Baena et al., 2014b
		*Cuspidaria*	Modified terminal leaflet	Sousa-Baena et al., 2014b
		*Tanaecium*	Modified terminal leaflet	Sousa-Baena et al., 2014b
		*Tynanthus*	Modified terminal leaflet	Lohmann, 2003
		*Pleonotoma*	Modified terminal leaflet	Lohmann, 2003
		*Lundia*	Modified terminal leaflet	Lohmann, 2003
		*Eccremocarpus*	Modified leaflets	D'Arcy, 1997
		*Tourrettia*	Modified leaflets	D'Arcy, 1997
	Plantaginaceae (2)	*Maurandya*	Twining petiole and pedicels	Fischer, 2004
		*Maurandella*	Twining petiole	Fischer, 2004
		*Epixiphium*	Twining petiole	Fischer, 2004
		*Lophospermum*	Twining petiole and pedicels	Fischer, 2004
		*Antirrhinum*	Twining pedicels	Fischer, 2004
Asterales	Asteraceae (4)	*Mutisia*	Modified terminal leaflet/prolonged rachis	Ulloa and Jørgensen, 1996; Hind, 2007
			Prolonged leaf tip	Hind and Hall, 2003b
			Prolonged leaf midrib	Hind and Hall, 2003a
		*Austrosynotis*	Twining petiole	Hind, 2007
		*Mikaniopsis*	Twining petiole	Hind, 2007
		*Cissampelopsis*	Twining petiole	Hind, 2007
	Campanulaceae (1)	*Canarina*	Twining petioles and pedicels	Lammers, 2007

*Numbers after family names represent the number of tendrils with different origins within that particular family. +, present. −, absent*.

**Figure 5:**
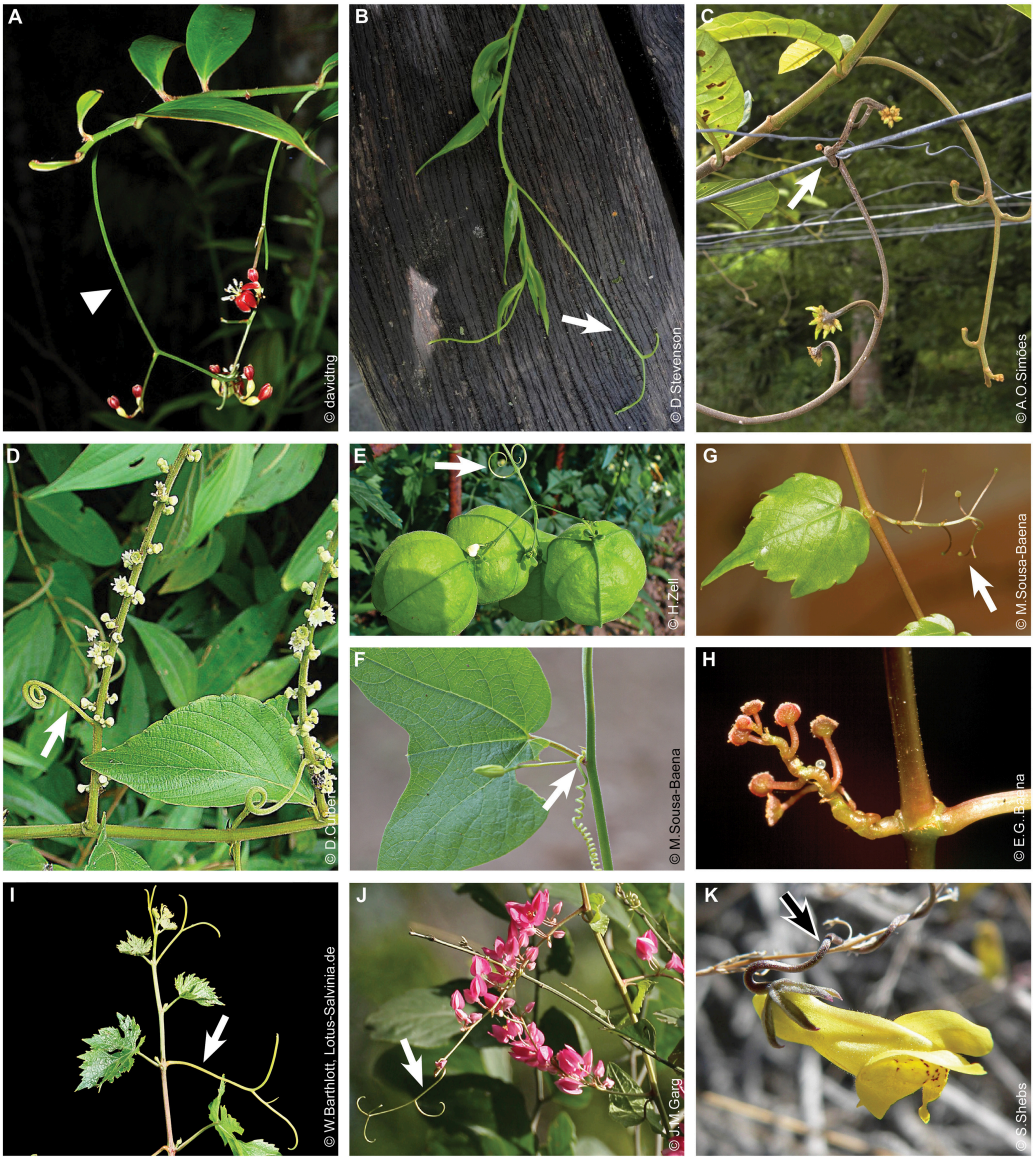
Tendrils derived from reproductive structures. **(A,B)** *Petermannia cirrosa* (Petermanniaceae), tendrils are modified inflorescences, note the similarity of the tendril in **(B)** with the inflorescence (arrowhead) in **(A)**. Image in panel **(A)** was reproduced with permission from [David Tng]. Image source: https://www.flickr.com/photos/davidtng. Image in panel **(B)** was reproduced with permission from [Dennis Stevenson]. Image source: http://www.plantsystematics.org/imgs/dws/r/Petermanniaceae_Petermannia_cirrosa_43766.html. **(C)** *Pacouria boliviensis*, tendril is a modified inflorescence rachis (Apocynaceae). This image was reproduced with permission from [André Simões]. Image source: http://floradobrasil.jbrj.gov.br. **(D–F)** Tendrils are modified inflorescence branches. **(D)** *Gouania lupuloides* (Rhamnaceae). **(E)** *Cardiospermum halicacabum* (Sapindaceae). **(F)** Tendril is originated from the tip of the reduced inflorescence apex in *Passiflora capsularis* (Passifloraceae). **(G–I)** Whole inflorescence modified into tendrils. **(G)** Young tendrils of *Parthenocissus tricuspidata* (Vitaceae), seen in their adult form, with terminal adhesive pads, in **(H)**. **(I)** *Vitis vinifera* (Vitaceae). This image was reproduced with permission from [Wilhelm Barthlott]. Image source: https://www.flickr.com/photos/lotus-salvinia. **(J)** Tendrils derived from the modified inflorescence tip in *Antigonon leptopus* (Polygonaceae). **(K)** Tendrils are modified pedicels in *Antirrhinum filipes* (Plantaginaceae). Arrows indicate tendrils.

Interestingly, tendrils derived from reproductive structures are found exclusively in the Core Eudicots (Figure 2). The only exception to that are the whole inflorescences modified into tendrils of *Petermannia cirrosa* (Petermanniaceae, Liliales) (Figures 5A,B; Conran and Clifford, 1998). Apart from the tendrilled vines and lianas found in the Asterales, not many other examples are found within asterid II. In this clade, stem climbers occur only in Pittosporaceae (Apiales) and Cardiopteridaceae (Aquifoliales). No lianas or vines are found in Escalloniales, Bruniales, Paracryphiales, and Dipsacales (Stevens, 2001 onwards). Hence, it seems that the ability to perform helical growth, and consequently its underlying developmental program, has been lost or modified in this angiosperms clade.

## The multiple ontogenetic origins of tendrils in angiosperms

Apart from having evolved multiple times, tendrils also have diverse ontogenetic origins. For instance, tendrils are considered to be modified inflorescences in grapes (*Vitis vinifera*, Vitaceae; Boss and Thomas, 2002) and several Passifloraceae (Feuillet and MacDougal, 2006). On the other hand, tendrils are modified leaflets in peas (*Pisum sativum*, Fabaceae; Hofer et al., 2009) and various members of tribe Bignonieae (Bignoniaceae; Sousa-Baena et al., 2014a,b). Tendrils are modified stems in pumpkins (*Cucurbita pepo*, Cucurbitaceae), cucumbers (*Cucumis sativus*, Cucurbitaceae) (Gerrath et al., 2008), and Sapindaceae (Acevedo-Rodríguez et al., 2011).

The ontogenetic origins of tendrils in different species are not always obvious. For instance, in Cucurbitaceae and Passifloraceae, although they are derived from different organs, tendrils are generated from a bud complex. Detailed developmental studies were necessary to identify from which portion of this complex structure tendrils were emerging. Even among foliar tendrils, it is tricky to identify the tendril origin. Even though it is currently known that tendrils derive from leaflets in peas (Hofer et al., 2009) and Bignoniaceae (Sousa-Baena et al., 2014a,b), they were thought to have been derived from modified rachises in representatives of these groups in the past. The difference may seem irrelevant in terms of morphology, but these two hypotheses are quite different in terms of molecular control. If a foliar tendril is derived from the rachis, drastic changes in left-right symmetry of the original organ would not be required in order to generate tendrils. On the other hand, if tendrils are derived from leaflets, this implies that a flat organ, with very intricate molecular regulation of medio-lateral and ab-adaxial symmetry establishment, would undergo significant changes in order to acquire a radialized symmetry.

Given that few anatomical or developmental studies are available to date, tendril origin is usually established solely based on organ position (i.e., topological correspondence) in most tendrilled families. Below, we summarize what is known regarding the development of tendrils with different ontogenetic origins (e.g., shoots, leaves and inflorescences) and their occurrence within angiosperms.

### Shoot derived tendrils

Several plant families have tendrils derived from modified shoots (Figure 4, Table 1), including the Annonaceae (Magnoliales) among the magnoliids; Connaraceae (Oxalidales), Passifloraceae (Malpighiales), Fabaceae and Polygalaceae (Fabales; Figure 3B), Rhamnaceae (Rosales), Cucurbitaceae (Cucurbitales; Figure 3A), and Sapindaceae (Sapindales) among the rosids; Olacaceae (Santalales), Icacinaceae (Icacinales), Apocynaceae, and Loganiaceae (Gentianales; Figure 3C) among the superasterids. Shoot branches acquiring tendril function evolved several times and are perhaps the most common tendril type within angiosperms (Table 1, Figure 2). Shoot derived tendrils are especially common among the rosids (Supplementary Figure 1A), in which practically all orders that contain tendrilled representatives (Celastrales, Oxalidales, Malpighiales, Fabales, Rosales, Cucurbitales, and Sapindales) have species bearing tendrils derived from shoots (Figure 2), with the exception of Brassicales and Vitales.

Tendrils are the most apparent morphological synapomorphy for Cucurbitaceae and are found in most species, except from a few taxa, where they have been transformed into thorns (e.g., *Momordica spinosa*) or lost (e.g., *Citrullus ecirrhosus* and *Melancium campestre*; Kocyan et al., 2007). In the Cucurbitaceae, tendrils develop in the leaf axils (Figure 3A) and have been thought to represent modified flowers (Darwin, 1875), leaves (Sensarma, 1955), and shoots (Sensarma, 1955; Gerrath et al., 2008). Detailed developmental studies of *Echinocystis lobata*, suggests that Cucurbitaceae tendrils are modified axillary shoots, with tendril ramifications corresponding to second-order branches (Gerrath et al., 2008). For a detailed review on the development and molecular regulation of tendrils in Cucurbitaceae see Sousa-Baena et al. (2018). To our knowledge, ontogenetic studies on shoot-derived tendrils have only been conducted on the Cucurbitaceae, and the exact origin and anatomical structure of such tendrils is unknown for all other plant families.

### Leaf derived tendrils

In many angiosperm families, whole leaflets or other leaf parts are modified into tendrils. Tendrils derived from leaflets are found exclusively within eudicots, occurring in Ranunculaceae (Tamura, 1993) and Papaveraceae (Kadereit, 1993), from Ranunculales, among the magnoliids; Fabaceae (Fabales; Figure 4D; Darwin, 1875) among the rosids; and Polemoniaceae (Ericales; Figure 4F; Wilken, 2004), Asteraceae (Asterales; Figure 4C; Hind, 2007), and Bignoniaceae (Lamiales; Figure 4E; Sousa-Baena et al., 2014a) among the asterids.

The most studied species with tendrils originated from leaves is *P. sativum* (pea, Fabaceae), as this species has historically been used as a model for studies in the field of genetics, experimental morphology and physiology. Pea leaves are composed of a pair of basal stipules and a rachis that exhibits up to three pairs of opposite leaflets, and up to three pairs of opposite leaflets modified into tendrils in the distal portion. Anatomical and molecular evidence support the hypothesis that *Pisum* tendrils are modified leaflets (Hofer et al., 2009). For a detailed review on development and molecular regulation of tendrils in *Pisum* see Sousa-Baena et al. (2018). The leaflets of the bipinnate leaves of *Entada scandens*, another Fabaceae, can also be modified into tendrils. However, tendril sensitivity to contact is different in *Entada*, as tendrils of *E. scandens* are sensitive over their entire surfaces, whereas *Pisum* tendrils are sensitive on their abaxial surface exclusively (Jaffe and Galston, 1968; Hofer and Noel Ellis, 2014). Other species of Fabaceae show different kinds of tendrils derived from other leaf parts. For instance, in *Lathyrus aphaca* the whole leaf is modified into a tendril, which is a unique case among angiosperms. In this case, photosynthesis is performed by the enlarged foliaceous stipules (Figure 3G; Sharma and Kumar, 2012).

Tendrils derived from modified leaflets are particularly common among asterids (Figure 2; Supplementary Figure 2A). They are found in the Ranunculaceae (Ranunculales; Tamura, 1993) and Papaveraceae (Ranunculales; Kadereit, 1993), magnoliids; and, Polemoniaceae (Ericales; Figure 4F; Wilken, 2004), Bignoniaceae (Lamiales; Figure 4E; Sousa-Baena et al., 2014a) and Asteraceae (Asterales; Figure 4C; Hind, 2007) in the asterids. A single study was conducted with a tendrilled species in Papaveraceae. This study documented intermediate stages between leaflets and tendrils in the leaf distal domain of *Cysticapnos vesicaria*, confirming the origin of tendrils in this family (Hidalgo et al., 2012). No data is available for other tendrilled species within Papaveraceae (see Table 1), or *Naravelia* (Ranunculaceae). However, some anatomical and ontogenetic studies are available for the Bignoniaceae and Asteraceae.

In Bignonieae, a large clade of lianas in the Bignoniaceae (Lohmann, 2006), leaves show great morphological variation. However, 2–3 foliolate leaves with the terminal leaflets modified into tendrils are the most common form (Lohmann and Taylor, 2014). Anatomical and molecular evidence support the hypothesis that tendrils are modified leaflets (Sousa-Baena et al., 2014a,b; for a review see Sousa-Baena et al., 2018). Foliar tendrils are also found in species of Tourrettieae, a small tribe of Bignoniaceae that includes four species with leaflet-derived tendrils (Gentry, 1980).

In Asteraceae, a large diversity of leaf tendril types are found in *Mutisia* (Cabrera, 1965), which has at least three tendrilling strategies. In the simple-leaved species, tendrils can be prolonged midribs (Figure 4B) or prolonged leaf tips (Figure 4A), such as those found in *Mutisia subspinosa*, which are somewhat similar to the tendrils of *Gloriosa* (Hind and Hall, 2003b). In species with prolonged midribs, the leaf lamina ends abruptly, forming a truncate leaf tip, and a thin tendril emerges from the midrib. In compound-leaved species, a simple or branched tendril (Figure 4C) develops at the tip of the rachis; this tendril type is described as a modified terminal leaflet by some authors, but others believe that these tendrils might actually represent modifications of the rachis. The exact origin of these tendrils will only be confirmed once developmental analyses of leaves are conducted in *Mutisia*.

Petiole-derived tendrils evolved several times and are one of the most common tendril types within angiosperms, occurring in many compound and simple-leaved families (Table 1, Figure 2). They are particularly common in herbaceous vines and among asterids (Supplementary Figure 1B). This condition is found in Hernandiaceae (Laurales), in the magnoliids; Menispermaceae, Ranunculaceae, and Papaveraceae (all Ranunculales), early eudicots; Tropaeolaceae (Brassicales; Figure 3F; Bulacio, 2013), in the rosids; and, Solanaceae (Solanales), Bignoniaceae (Lamiales), and Asteraceae (Asterales), in the superasterids. In various species in which this tendril type is found, petioles begin to function exclusively as a tendril after clasping the support, with the leaf blade undergoing abscission, as is observed in *Perianthomega vellozoi* (Bignoniaceae). Twining petioles can sometimes become remarkably elongated and thickened, as observed in species of *Solanum* from the Dulcamaroid clade (Knapp, 2013). Many species that have twining petioles also show twining rachises, e.g. species of *Clematis* (Ranunculaceae) and *Cysticapnos* (Papaveraceae). Although tendrils derived from twining petioles have evolved numerous times within angiosperms, they are typical of some taxa, such as *Tropaeolum* (Brassicales; Figure 3F; Bulacio, 2013).

The opposite situation is rare, i.e., for an organ, after functioning as a tendril, to acquire other functions. This is the case of *Nepenthes* (Nepenthaceae, Caryophyllales) though, in which the tip of the basal laminar portion first develops as a slender coiling tendril used for twining around supporting structures. These tendrils are transitory structures, as their tips subsequently develop into a pitcher (Figure 3E; Franck, 1976). The most accepted theory to explain this complex structure is based on morphology and proposes that the pitcher found in *Nepenthes* is a modified peltate leaf, in which the flattened basal portion (photosynthetic) corresponds to the base of regular leaves. In this genus, the tendril actually corresponds to a modified petiole, while the cup is a highly modified lamina (Franck, 1976). *Triphyophyllum peltatum* (Dioncophyllaceae), another carnivorous plant from Caryophyllales, has tendrils originated from the leaf apical region. Tendrilled leaves develop at maturity, after the plant has become a vigorous climber. These leaves have apical hook-like tendrils (Figure 3H) that are used for climbing. After touching a suitable support, the tendrils acquire a limited helicoidal growth and grasp it (Figure 3H; Grenn et al., 1979). These tendrils are apparently formed by the elongation and bifurcation of the midrib (Takhtajan, 1997).

Foliar tendrils are also found among monocots, more specifically in Liliales (Liliaceae and Colchicaceae), Asparagales (Asparagaceae), and Poales (Flagellariaceae; Table 1, Figure 2). In *Fritillaria* (Liliaceae), *Gloriosa* and *Littonia* (Colchicaceae; Figure 3I), *Flagellaria* (Flagellariaceae), and *Polygonatum* (Asparagaceae), the leaf apex is elongated and attenuated into a recurved coiling tendril that twines around other vegetation (Arber, 1920; Nordenstam, 1998). In particular, *Flagellaria* tendrils may coil vigorously and become quite thickened in their adaxial portion (Hesse et al., 2016). Tendrils similar to the ones observed in these groups are found in some species of *Mutisia* (Asteraceae, core eudicots), representing an interesting case of convergent evolution (Supplementary Figure 2B). Comparative studies analyzing the degree of tendril similarity at the anatomical level among these taxa are still lacking though.

An interesting tendril type is found in *Smilax* (Smilacaceae, Liliales), which has a pair of tendrils that is associated with the leaf petiole (Figure 3D). Such tendrils have been interpreted as derived from stipules, leaflets, thorns, trichomes, or emergences, and as duplications of the petiole (Arber, 1920; Conran, 1998). The co-occurrence of stipules and tendrils within a single species, associated with a lack of ternate leaves in the family indicates that tendrils could not be derived from stipules or leaflets in this genus though (Conran, 1998). Furthermore, anatomical evidence on leaf and tendril vasculature refuted the hypothesis that tendrils are derived from thorns, trichomes or emergences (Arber, 1920). Smilacaceae tendrils apparently originated from petiole duplication. As such, each tendril would be equivalent to a petiole plus a pseudo-lamina (Arber, 1920). This interpretation adopts the phyllode theory, and states that tendrils are generated through chorisis, i.e., the separation of an organ into two or more parts by division during development. Hence, *Smilax* tendrils would be homologous to petioles (Arber, 1920).

### Tendrils derived from reproductive structures

Tendrils derived from modified reproductive structures are found in many angiosperm families (Figure 2, Table 1), including the Petermanniaceae (Liliales; Figures 5A,B) among the monocots; Vitaceae (Vitales; Figures 5G–I), Passifloraceae (Malpighiales; Figure 5F), Rhamnaceae (Rosales; Figure 5D), Sapindaceae (Sapindales; Figure 5E), and Tropaeolaceae (Brassicales) among the rosids; Polygonaceae (Caryophyllales; Figure 5J), Apocynaceae (Gentianales; Figure 5C), Plantaginaceae (Lamiales; Figure 5K) and Campanulaceae (Asterales) in the superasterids.

The most studied taxa are Vitaceae and Passifloraceae (Malpighiales). In Vitaceae tendrils are opposite to leaves and can be simple or branched (Figures 5G–I; Wen, 2007). The tendrils of Vitaceae develop from a meristematic structure called “uncommitted primordium,” which is a meristematic structure that develops at the SAM, already opposite to the leaf primordium since its inception. After initiation, the uncommitted primordium usually forms a main and a lateral arm that have independent developmental fates (Gerrath et al., 2017). Depending on environmental and internal cues, the uncommitted primordium will either develop into a tendril, or into an inflorescence, or an intermediate organ between the two, i.e., a tendril/inflorescence hybrid structure (Gerrath et al., 2015). For instance, in *Ampelopsis*, the hybrid organ usually has a terminal flower or flowers, and the tendril/inflorescence rachis maintains its coiling ability (Gerrath and Posluszny, 1989; Gerrath et al., 2017). In *Rhoicissus*, the inflorescences may be a tendril/inflorescence hybrid with flowers being formed only in the main arm, or in both arms (Gerrath et al., 2004, 2017). Such structures are also found in *Cissus antarctica* (Gerrath and Posluszny, 1994), *Pterisanthes eriopoda* (Ickert-Bond et al., 2015), and are diagnostic for *Ampelocissus* (Gerrath et al., 2015). In *Vitis*, usually only tendrils are generated in young plants, and tendril or inflorescences in adult plants (Tucker and Hoefert, 1968; Wen, 2007). However, it is not uncommon for the inner arm of the uncommitted primordium to generate an inflorescence and the outer arm to develop into a tendril in adult plants of this genus (Gerrath et al., 2015). Interestingly, experiments with *V. vinifera GA-insensitive* mutant plants (*Vvgai1*), in which tendrils are replaced by inflorescences, concluded that tendrils could be considered modified inflorescences inhibited from completing their development by GAs (Boss and Thomas, 2002). In addition, experiments using exogenous GA in *V. vinifera* showed the uncommitted primordium would rather develop into a tendril in the presence of such plant hormones (Srinivasan and Mullins, 1980). Hence, results from morphological, physiological and molecular developmental analyses of *V. vinifera* tendrils strongly suggest that they are modified inflorescences (Gerrath et al., 2017).

Many species of Passifloraceae have tendrils (Feuillet and MacDougal, 2006) located in the leaf axils. The link between tendril and reproductive development is well-known for some species in which tendrils are not produced in juvenile plants, but start to develop after transition to the adult phase (Gangstad, 1938; Nave et al., 2010; Cutri et al., 2013). In addition, ontogenetic and molecular studies also suggest that tendrils originate from modified reproductive shoots and represent the modified distal portion of the inflorescence (Prenner, 2014). For a detailed review on development and molecular regulation of tendrils in Vitaceae and Passifloraceae see Sousa-Baena et al. (2018). Vitaceae tendrils with terminal adhesive pads are discussed in the following section.

Many other families of angiosperms have tendrils associated with reproductive structures. However, tendril developmental studies are lacking for these families. This is the case for several species of Sapindaceae (Sapindales; Figure 5E), a family with many tendrilled genera (e.g., *Lophostigma, Cardiospermum*, and *Paullinia*). However, virtually no developmental studies are available on tendril development for this plant family. These three genera have a pair of tendrils at the base of the inflorescence axis (Figures 5E,H; Acevedo-Rodriguez, 1993; Acevedo-Rodríguez et al., 2011). Studies on the development of tendrils in *Cardiospermum halicacabum* showed that both tendrils are opposite axillary branches of the inflorescence, i.e., tendrils are homologous to lateral floral branches (Dave and Shah, 1971). Two or three accessory buds are associated with the inflorescence axils, of which one develops into a tendril, while the remaining buds are dormant. The fact that only one tendril develops at the base of the cyme, while the opposite branch bears flowers and looks like an inflorescence branch (Dave and Shah, 1971), further supports an inflorescence origin for tendrils in *C. halicacabum*.

Inflorescences modified into tendrils are also found in Polygonaceae (Caryophyllales; Figure 5J; Shah and Dave, 1971b) and Rhamnaceae (Rosales; Figure 5D; Medan and Schirarend, 2004). In *Antigonon leptopus* (Polygonaceae; Figure 5J) the inflorescences are axillary racemes, slightly branched, bearing lateral tendrils mainly on their distal portions. The main axis of the inflorescence terminates in two or three tendrils (Shah and Dave, 1971b). *Antigonon* tendrils have been thought to represent modified inflorescence apices and axis. In this species, bracts and tendrils bear fascicles of flowers in their axils during the flowering period. However, axillary buds develop into a main tendrillar axis with 3-4 leafy lateral bracts, 2–3 lateral and 2–3 terminal tendrils, even during the vegetative phase (Shah and Dave, 1971b). Developmental analysis revealed that histological inception of tendrils in *A. leptopus* is similar to that of bract primordia. In addition, floral buds arise in the axils of both bracts and tendrils during the flowering season, corroborating the hypothesis that lateral tendrils in the inflorescence axis are modified bracts (Shah and Dave, 1971b). It has also been shown that the seemingly terminal tendrils of this species are in fact lateral, since a vestigial apical meristem may still be recognized for a short period after their initiation (Shah and Dave, 1971b). In Rhamnaceae, three kinds of tendrils exist. In *Gouania*, tendrils may originate from the inflorescence rachis (representing a modified branch) and peduncle; tendrils derived from stem modifications can also be found in the same genus (Tortosa, 2005). Two other cases of tendrils derived from reproductive structures occur in *Pacouria* (Apocynaceae; Gentianales; Figure 5C), and in *Antirrhinum* (Plantaginaceae; Lamiales; Figure 5K). In the former, the inflorescence main axis acquires a climbing function (Simões et al., 2007), in the latter the flower pedicel performs this function (Fischer, 2004). Interestingly, the tendrils terminated in flowers of *Ampelopsis* (Vitaceae) have been interpreted as an intermediate structure between tendrils and inflorescences, but they could in fact be considered as a tendril derived from an inflorescence rachis that acquired the capacity for helical growth, becoming voluble, as observed in *Pacouria* (Apocynaceae; Figure 5C).

Within monocots, whole inflorescences modified into tendrils are found in Petermanniaceae (Liliales; Takhtajan, 1997; Figures 5A,B). In *P. cirrosa*, tendrils are leaf-opposed and branched. The inflorescences and tendrils are terminal, but become leaf-opposed when they are displaced by the vigorous growth of an axillary shoot (Tomlinson and Ayensu, 1969). Evidence that supports the homology between tendrils and whole inflorescences, include their identical position as inflorescences, the fact that both have branches subtended by scale-like bracts, and the fact that the inflorescence rachis and tendrils are anatomically identical (Tomlinson and Ayensu, 1969). Furthermore, although not formally published, a tendril derived from a notably elongated inflorescence rachis, which has the capacity for helical growth (as in *Pacouria*), has been observed in *Oncidium* sp. (Orchidaceae, Asparagales; Wilhelm Barthlott, *pers. comm*.; Supplementary Figure 3).

## A special case: convergent evolution of tendrils with adhesive pads

The ability of tendrilled plants to climb is inversely proportional to the diameter of the support (Putz and Holbrook, 1991). On the other hand, this limitation does not exist in plants that develop tendrils with adhesive tips because the adhesive pads can attach to vertical supports independently of their overall size and shape. Tendrils with adhesive pads or disks at their tips evolved solely in Vitaceae (Vitales), Passifloraceae (Malpighiales), Bignoniaceae (Lamiales), and Cucurbitaceae (Cucurbitales). These are distantly related families that include large numbers of scandent taxa. The ontogenetic origin of tendrils, mechanism of tendril attachment, and adhesive pad morphology vary substantially among those families.

In Vitaceae, the tendril tips of *Parthenocissus tricuspidata* (Boston Ivy; Figures 5G,H) and *Parthenocissus quinquefolia* (Virginia Creeper) develop nearly spherical adhesive pads that attach firmly to varied surfaces. Like the non-adhesive tendrils found in other Vitaceae, adhesive tendrils originate from modified lateral branches in *Parthenocissus*. These branches have ramifications, each one ending in a swollen, round tip that develops into a flattened adhesive pad upon contact with a suitable substrate (Steinbrecher et al., 2011). The adhesive pad grows toward the substrate, but unlike other lineages with adhesive tendrils, species of *Parthenocissus* do not show morphologically different pads whose growth is molded by the substrate topography (Steinbrecher et al., 2011). After contact, the epidermal cells of the tendril tips elongate toward the substrate, becoming papillate. These papillate cells undergo changes in shape that mimic the topography of the support, whereas the pad remains circular in shape. Adhesive fluid helps to fill the crevices of the substrate, so that a perfect form closure between the attachment pads and the substrate is formed (Bowling and Vaughn, 2008; Steinbrecher et al., 2011). The adhesive fluid forms a peripheral ring around the adhesive pad and it is composed of rhamnogalacturonan I, callose, and other mucilaginous pectins (Bowling and Vaughn, 2008). Adhesive tips also occur in young plants of some species of *Cissus* and in *Tetrastigma obtectum* (Wen, 2007).

Adhesive pads also evolved in *Passiflora* (Passifloraceae). Most species of *Passiflora* are vines that climb through axillary tendrils (Shah and Dave, 1971a; Feuillet and MacDougal, 2006). In most species, tendrils are simple and perform contact coiling when attaching to a support. Nevertheless, some species from the subgenus *Deidamioides* show branched tendrils with terminal adhesive pads, a feature that is exclusive to this subgenus (Krosnick et al., 2013). This is the case in three species of *Passiflora*, i.e., *Passiflora discophora, P. arbelaezii*, and *P. tryphostematoides* (Bohn et al., 2015). Tendrils of *P. discophora* are 3-to-5 branched with terminal adhesive pads. A recent developmental analysis of *P. discophora* revealed that tendrils perform contact coiling that is combined with the development of adhesive pads for climbing (Bohn et al., 2015). Before initial contact with a substrate, tendril apices are slightly hook shaped. After contact, the epidermal cells in the tendril tip become papillate. The proliferation of papillate cells leads to the formation of the pad, with a porous surface. This porous pad tissue develops at the tendril tips and along portions of the tendril axes that are in contact with the substrate (Bohn et al., 2015). Differently from *Parthenocissus*, the pads of *P. discophora* tendrils adapt to the topography of the substrate, and thus have varied shapes; when growing on flat supports they have a hemispherical shape. *P. discophora* also produces an adhesive fluid, that is made of cutin and lipids, and mainly concentrated in the central part of the contact zone (Bohn et al., 2015). The fluid enhances the form closure by filling micro- and nanogaps and intercellular spaces in the contact zone (Bohn et al., 2015).

In tribe Bignonieae (Bignoniaceae, Lamiales), tendrils with adhesive pads are found in *Amphilophium, Mansoa parvifolia, Manaosella cordifolia*, and *Bignonia capreolata*, all of which bear parted, i.e., trifid or multifid, tendrils (Lohmann and Taylor, 2014). Different from Vitaceae, the tips of adhesive tendrils of members of tribe Bignonieae do not show small immature pads before reaching the substrate, being more similar to tendrils of *Passiflora* in this respect (Darwin, 1875; Sousa-Baena et al., 2014a). The attachment mechanism of *B. capreolata* tendrils was studied in detail by Darwin (1875), who observed that tendril tips develop into irregularly shaped attachment pads once they contact suitable substrates. These pads grow into surface crevices and excrete a resin-like substance that serves as glue, reinforcing the attachment (Darwin, 1875). Prior to contact, tendril tips of *B. capreolata* were described as blunt hooks (Darwin, 1875), while those of *Amphilophium crucigerum* were described as hooked (Seidelmann et al., 2012). However, developmental studies of *Amphilophium buccinatorium* and *A. crucigerum* showed that tendril tips bear a flattened structure, similar in cross section to leaflet primordia (Sousa-Baena et al., 2014a,b). This small leaflet-like projection has a curvature in relation to the tendril stalk giving a hook-like aspect to tendril tips.

In tribe Bignonieae, the ability to produce adhesive pads at the tips of tendrils seems to be correlated to a longer maintenance of meristematic state during leaf development, enabling the generation of branched tendrils at the leaf distal portion, as well as the development of adhesive pads at the tendril tip, which are derived from the leaf-like structure found in young tendril tips (Sousa-Baena et al., 2014a). Developmental studies of adhesive tendril tips in *A. crucigerum* showed that their pads grow similarly to those of *B. capreolata*, but do not excrete any glue-like substance (Seidelmann et al., 2012). In *A. crucigerum*, only tips that touch a rough substrate develop into pads, indicating that the initiation of pad development is triggered by specific contact stimuli (Seidelmann et al., 2012). Interestingly, when two or more pads grow next to each other, they may merge into one large attachment structure (Seidelmann et al., 2012). Multicellular papillae are found in the adaxial portion of the tendril leaf-like tip, during the initiation of the pad; this papillary tissue contains phenolics and grows into gaps and crevices of the support surface, filling them completely (Seidelmann et al., 2012).

Some species of Cucurbitaceae have tendrils with adhesive pads, apparently similar to those of *Parthenocissus* (Vitaceae). This is the case in *Alsomitra macrocarpa, Bayabusua clarkei, Neoalsomitra sarcophylla, Polyclathra cucumerina*, and *Trichosanthes cucumerina* (Kocyan et al., 2007). To our knowledge, developmental studies on tendril adhesive pads are lacking for Cucurbitaceae, as well as their mode of attachment. It is also unknown whether glue-like substances are secreted by Cucurbitaceae tendril pads.

Some common morphological characteristics are found in all adhesive tendrils, regardless of their origin and phylogenetic placement. Examples of those are the tendril ramifications, and main and secondary axis possessing the ability to perform free coiling (Bohn et al., 2015). Interestingly, adhesive pads seem to occur much more frequently in branched tendrils. As such, it is possible that genes related to the prolonged maintenance of the meristematic state are involved in the development of branched tendrils and apical pads outside *Amphilophium*. The only exception was found in *Passiflora obovata*, which bears simple tendrils and climbs using apical adhesive pads during juvenile stages (Krosnick et al., 2013). All species analyzed so far have elongated epidermal papillate cells at the interface between the adhesive pads and the substrate. During growth, these cells are able to conform to the surface irregularities by fixing the pad in minute fissures of the substrate, forming a close mechanical interlock (form closure). While *Parthenocissus* pads always remain circular, those of *Passiflora* and *Amphilophium* can vary depending on the substrate. Moreover, the form closure can be enhanced by glue-like extracellular substances that act as filler in *Parthenocissus, P. discophora*, and *B. capreolata*, but not *Amphilophium*. As such, the internal structure of the adhesive pads seems to differ among species, and may be associated with the morphological origin of tendrils (Bohn et al., 2015).

## Summary of progress and avenues for future research on tendril biology

Studies in the field of developmental molecular genetics have shed light on tendril origin in some plant families. The molecular basis of tendril development has been investigated in Fabaceae (mainly in *P. sativum*, but also in *Lens* and *Lathyrus*), Vitaceae (mainly in *Vitis*), Cucurbitaceae (in *C. sativus* and *Cucumis melo*), Passifloraceae (in *Passiflora edulis*) and Bignoniaceae (in some species of the tribe Bignonieae). Tendrils in these lineages do not share many developmental characteristics, and have diverse ontogenies that seem to be regulated by different genetic networks (Sousa-Baena et al., 2018).

For instance, the development of leaflet-derived tendrils in *P. sativum* is controlled by *LEAFY/FLORICAULA* (*LFY/FLO*; Hofer et al., 1997), through interactions with *TENDRILLESS* (*TL*), a Class I homeodomain leucine zipper (HD-ZIP) gene (Hofer et al., 2009), and *LATHYROIDES* (*LATH*), a *WUSCHEL-related homeobox1* (*WOX1*) transcription factor (Zhuang et al., 2012). On the other hand, the development of leaflet-derived tendrils from representatives of the tribe Bignonieae is controlled by *SHOOTMERISTEMLESS* (*STM*), a *KNOX1* gene, likely through the interaction with *PHANTASTICA* (*PHAN*), an *ARP* (*asymmetric leaves1*/*rough sheath*/*phantastica*) gene (Sousa-Baena et al., 2014a). Noteworthy, studies of *CRISPA*, the *PHAN* ortholog in *P. sativum*, showed that tendril development was not altered in *CRISPA* mutants (Tattersall et al., 2005). Furthermore, *LFY/FLO, APETALA1* (*AP1*) and *FRUITFULL* (*FUL*) transcription factors, known for being involved in the reproductive development of *Arabidopsis thaliana*, are expressed during tendril development in *P. edulis* (Cutri, 2009; Scorza et al., 2017) and *V. vinifera* (Carmona et al., 2002; Calonje et al., 2004). However, *LFY/FLO* is expressed in tendril meristems of *V. vinifera*, but only in tendril tips at more advanced developmental stages in *P. edulis* (Cutri, 2009). *AP1* has a similar expression pattern only in tendril primordia and tendrils at early developmental stages. The expression of *AP1* is maintained in the whole organ in *P. edulis*, but only in tendril arms and branching zone in *V. vinifera*. *FUL* is expressed in all developmental stages of *P. edulis* tendrils (Scorza et al., 2017), but only at early stages during the development of *V. vinifera* tendrils (Calonje et al., 2004).

Such differences in molecular control simply reflect that tendrilled species belong to distantly related lineages and have diverse ontogenies. *P. sativum* is from the Fabaceae family, which belongs to the order Fabales, in the rosid I clade, while the Bignoniaceae belongs to the order Lamiales, placed in the asterid I clade. In *P. sativum* leaf development is complex, with the lateral organs being initiated in an acropetal sequence, while the organs in the leaf distal domain develop basipetally (Hofer and Ellis, 1998). In Bignonieae, leaves develop acropetally throughout (Sousa-Baena et al., 2014a,b). *V. vinifera* and *P. edulis* belong to the rosids and are more closely related to each other than to *Pisum* or Bignonieae species. However, tendrils seem to be modified whole inflorescences in Vitaceae (Gerrath et al., 2017), but originated from the tip of a reduced inflorescence apex in Passifloraceae (Prenner, 2014). Also, *V. vinifera* tendrils develop from an uncommitted primordium, which has a structural organization similar to that of the SAM (Tucker and Hoefert, 1968). On the other hand, *P. edulis* tendrils develop from tendril buds with no discernible histological zonation (Shah and Dave, 1971a). These findings indicate that a common organ origin does not necessarily imply that tendrils will share similar developmental pathways, or be controlled by the same genetic networks.

Recent studies on inflorescence development in Vitaceae, using a broad sampling of taxa described intermediate forms between tendril and inflorescence in various genera (Gerrath et al., 2017). Furthermore, *AP1, FUL*, and *FLOWERING LOCUS T* (*FT*) orthologs were shown to be expressed in tendrils of five other genera of Vitaceae, besides *Vitis* (Zhang et al., 2015). The existence of these hybrid structures, and the fact that genes known to control reproductive development are expressed in tendrils of various genera of Vitaceae, strongly suggests that tendrils are homologous to inflorescences in this family (Gerrath et al., 2017). However, this hypothesis still needs to be tested through functional studies. These studies will be important in order to demonstrate that tendrils indeed represent modified inflorescences in Vitaceae. Furthermore, those studies should also lead to a better understanding of the inflorescence/tendril hybrid structures.

Recent molecular studies have also corroborated the ontogenetic origin of tendrils in Cucurbitaceae. Tendril-less lines of *C. sativus* and *C. melo* have been identified (Mizuno et al., 2015; Wang et al., 2015), in which tendrils are replaced by lateral shoots, demonstrating that tendrils are modified lateral branches in those species. This phenotype is caused by *TEN* in *C. sativus*, and its homolog in *C. melo, CmTCP1*, from the *TEOSINTE BRANCHED1, CYCLOIDEA and PROLIFERATING CELL FACTORS1/2* (*TCP*) gene family (Mizuno et al., 2015; Wang et al., 2015). Another *C. sativus* tendril-less mutant has been recently described, *tendril-less1* (*td-1*; Chen et al., 2017). *Tendril-less1* mutants lack tendrils and this structure is not replaced by any other organs, a phenotype that is caused by the mutation in the gene *CsGCN5* (*C. sativus GENERAL CONTROL NONDEREPRESSIBLE 5*; Chen et al., 2017). Climbing plants are able to locate their support and grow toward them, thus actively growing toward the dark. This behavior is known as skototropism (Gianoli, 2015). Interestingly, *CmTCP1/TEN* belongs to the same clade as *TEOSINTE BRANCHED1-like* (*TB1-like*), whose expression is induced by shade/dark condition, and then negatively regulates axillary bud outgrowth (Nicolas and Cubas, 2015). Also, *A. thaliana AtGCN5* is associated with light-activated expression of various genes (Li et al., 2012). Hence, it is possible that these genes may be involved in light signaling during the skototropic behavior of shoot-derived tendrils in Cucurbitaceae.

All tendrils perform helical growth, which is an interesting case of functional convergence. The ability to perform helical growth is independent from phylogenetic history or ontogeny. In a taxonomically broad survey, tendrils that coiled in many different directions were shown to have a cylinder of cortical gelatinous fibers (Meloche et al., 2007; Bowling and Vaughn, 2009), whereas tendrils that coiled only in a single direction were shown to only have gelatinous fibers along the inner surface of the coil (Bowling and Vaughn, 2009; Gerbode et al., 2012). These fibers play a central role in tendril function in *Cucumis* sp., as the ribbon-like strip of gelatinous fiber cells retains its helical shape when extracted from the coiled tendril, suggesting that it is the shaping of the fiber strip that drives the coiling of the tendril soft tissues (Gerbode et al., 2012). These data suggest that coiling and twining in vines may be caused by gelatinous fibers (Bowling and Vaughn, 2009; Gerbode et al., 2012). However, isolated cells growing in culture may acquire helical shape (Buschmann et al., 2009), demonstrating that helical growth is not exclusively related to multicellular structures, or to the anatomy of tendril and twining stems. Indeed, helical growth seems to be enabled by a more universal mechanism of cell shaping involving the cytoskeleton.

Most plant cells expand anisotropically, i.e., along a preferred axis. The orientation of cell expansion is maintained by the cortical microtubule cytoskeleton, which guides the deposition of cellulose microfibrils in specific direction in the cell wall (Paredez et al., 2006). Hence, polarity of cell expansion, plant growth and organ formation are intimately linked to cytoskeletal components (Wada, 2012). Numerous genes are responsible for keeping cell orientation straight, and left-right asymmetry can be induced by mutation. Several *A. thaliana* mutants in which roots, hypocotyls, petioles and inflorescence stems are twisted are known (Ishida et al., 2007). Studies with *A. thaliana spiral* right-hand and *lefty* left-handed mutants, showed microtubules are directly involved in the helical growth observed in such phenotypes (Furutani et al., 2000; Thitamadee et al., 2002). There is a striking similarity between twining stems of wild climbers and the inflorescence stems of the *spr1-4 sp1l3-1 A. thaliana* double mutants that twine around support forming a right-handed helix. In wild-type *A. thaliana* plants, axial organs maintain straight expansion, and the plant cell cortical microtubule array is aligned transversely to the primary growth axis (Wada, 2012). In contrast, the growth direction of the cells in the twisting phenotypes is tilted either to the right or to the left, a pattern that results from an oblique orientation of the microtubules that have helical arrangement (Paredez et al., 2006). Hence, it is likely that at least some of the genes associated with helical growth in *A. thaliana* mutants are linked to the helical growth of shoots and tendrils in wild climbers.

## Conclusions

Climbers are abundant in tropical rainforests, accounting for a large percentage of the floristic diversity encountered in those forests. The acquisition of the climbing habit in flowering plants constitutes a key innovation. Highly diversified families of climbing plants usually have other climbing mechanisms in addition to stem twining, such as tendrils, i.e., specialized organs with threadlike shape that twine around other structures through helical growth, representing an efficient climbing strategy. Even though angiosperms have a large number of tendrilled representatives, the development of tendrils has only been studied in a few species, leaving an immense knowledge gap in the biology of such organisms. Studies in developmental molecular genetics have been shedding light into tendril origin. For instance, developmental molecular genetic studies provided key insights into the origin of tendrils in Cucurbitaceae. On the other hand, such studies have also shown that tendrils from various phylogenetic lineages have diverse ontogenies that are regulated by different gene networks, even when derived from the same organ.

In this context, future research on the biology and evolution of tendrils should include ontogenetic and molecular studies for all tendril types within angiosperms, specially targeting distantly-related lineages with tendrils derived from the same organ. For instance, functional studies of leaflet-derived tendrils of Bignonieae would allow a proper comparison of tendrils in this tribe with those from *P. sativum*. Furthermore, functional studies targeting floral genes in Vitaceae and Passifloraceae would clarify the exact origin of tendrils in these families, probably elucidating the mechanism by which the inflorescence/tendril hybrid structures are formed in Vitaceae. In addition, investigating the role of cytoskeletal components in the helical growth of tendrils in wild climbers could shed light on the mechanisms that enabled such widespread functional convergence in angiosperms. Interestingly, tendrilled climbers are more prominent in early successional environments and disturbed forest types characterized by thinner host stem diameters. Owing to the weedy nature of some tendrilled species, these taxa can become serious invaders, causing economic problems. As such, it is important that new research on the biology of tendrilled species is conducted so an improved understanding and management of these plants can be achieved. Such efforts are particularly important given the increasing environmental disturbance in the tropics.

## Author contributions

MS-B, LL, and NS conceived the ideas; MS-B compiled most of the information and led the writing; JH-L led the writing of the section about tendrils derived from reproductive structures; LL and NS contributed with writing in various sections. All authors revised the manuscript extensively.

### Conflict of interest statement

The authors declare that the research was conducted in the absence of any commercial or financial relationships that could be construed as a potential conflict of interest.
