# Supplementary material for: Convergent Evolution and the Diverse Ontogenetic Origins of Tendrils in Angiosperms
Source: Front Plant Sci. 2018 Apr 3;9:403. doi: 10.3389/fpls.2018.00403 (PMC5891604; doi:10.3389/fpls.2018.00403)
Supplement: Supplementary file 1 [file Image1.PDF]

Supplementary Material

Convergent Evolution and the Diverse Ontogenetic Origins of Tendrils in Angiosperms

Mariane S. Sousa-Baena*, Neelima R. Sinha, José Hernandes-Lopes, Lúcia G. Lohmann

*** Correspondence:** Mariane S. Sousa-Baena: m.sousabaena@yahoo.com

1. Supplementary Figures

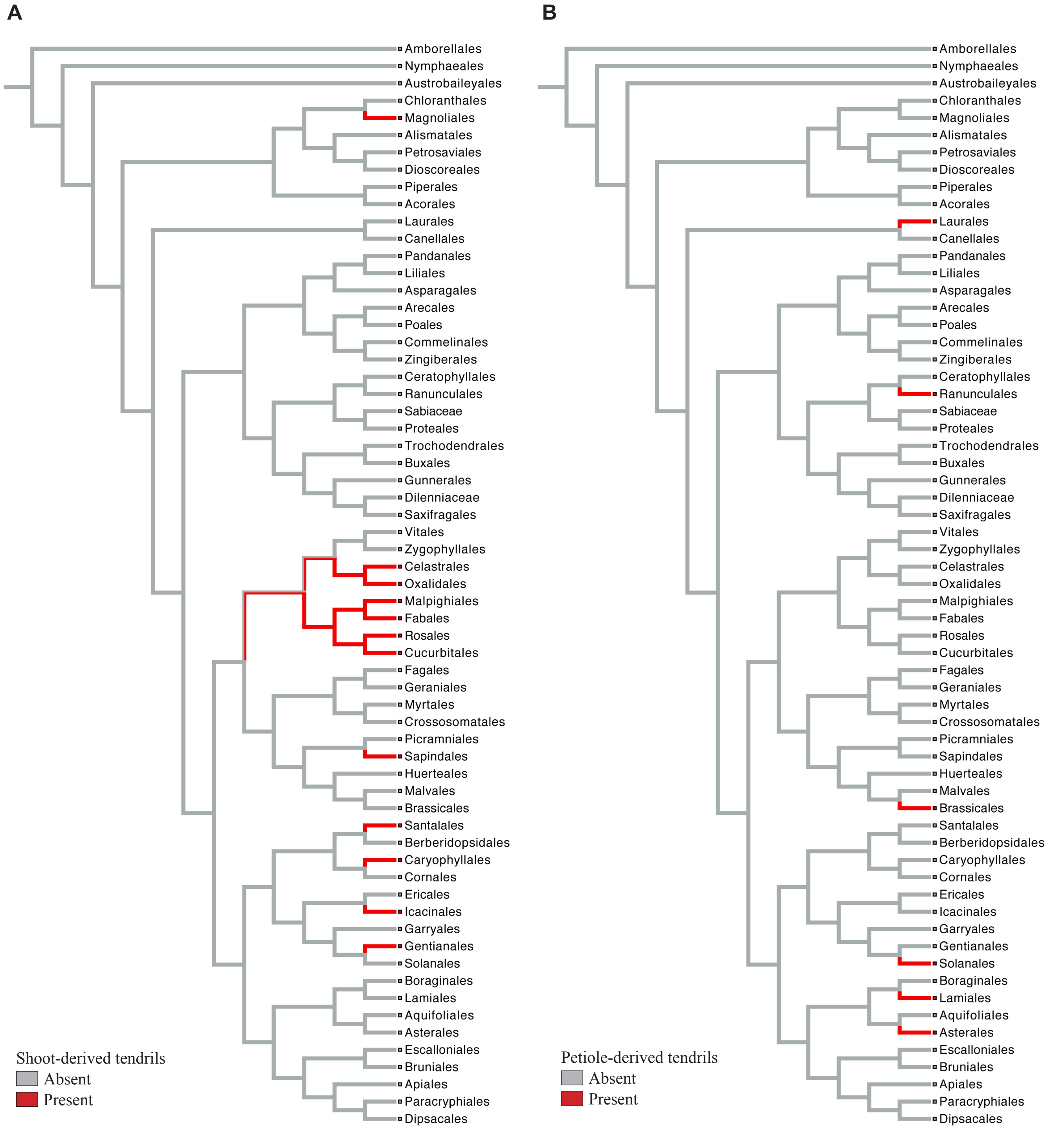

Supplementary Figure 1. Angiosperm phylogeny modified from Stevens (2001 onwards) to include Icacinales (following the APG IV; The Angiosperm Phylogeny Group, 2016), with parsimony ancestral state reconstruction of tendrils derived from modified shoots (A) and from modified petioles (B).
