# Supplementary material for: Convergent Evolution and the Diverse Ontogenetic Origins of Tendrils in Angiosperms
Source: Front Plant Sci. 2018 Apr 3;9:403. doi: 10.3389/fpls.2018.00403 (PMC5891604; doi:10.3389/fpls.2018.00403)
Supplement: Supplementary file 2 [file Image2.PDF]

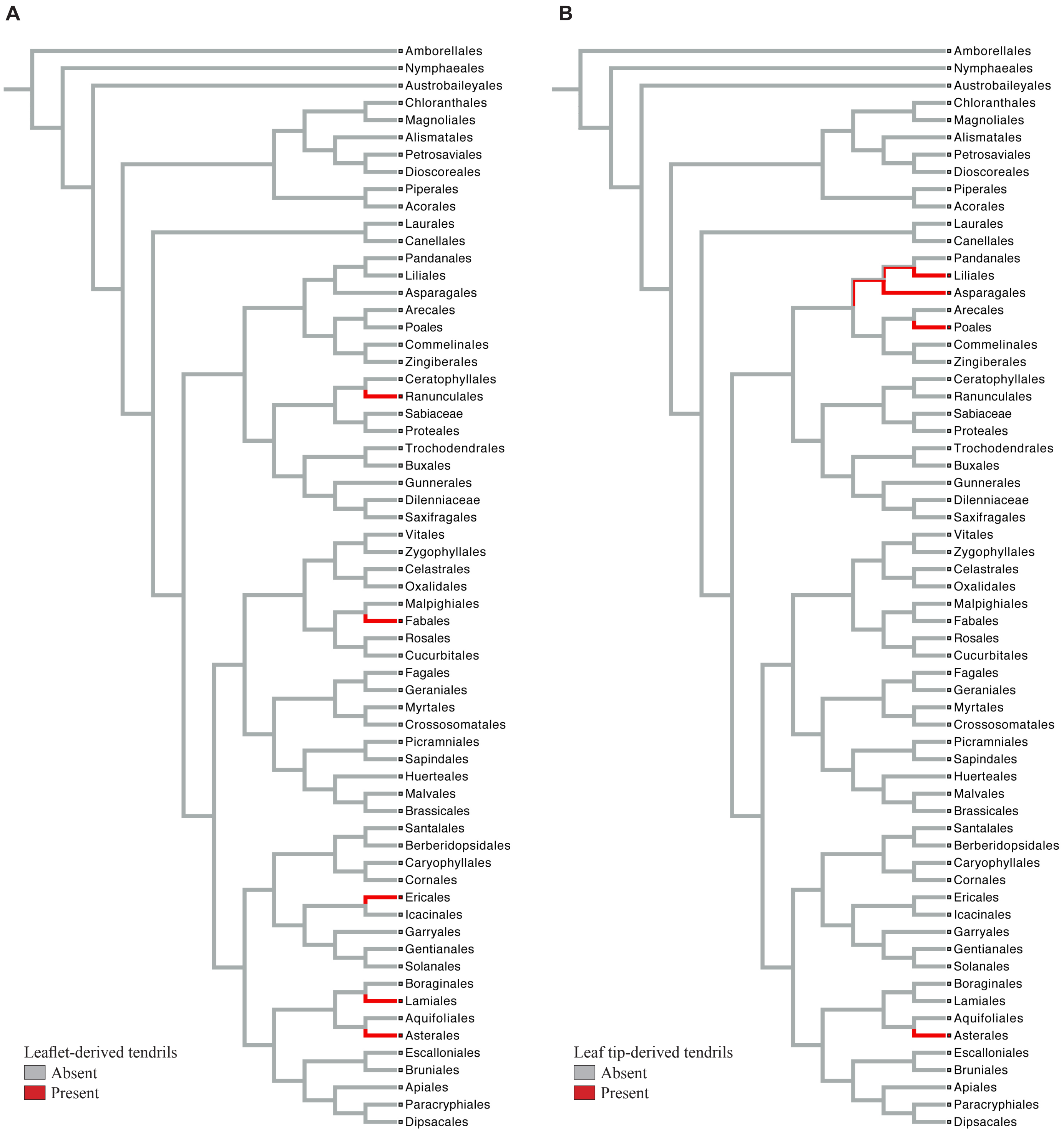

Supplementary Figure 2. Angiosperm phylogeny modified from Stevens (2001 onwards) to include Icacinales (following the APG IV; The Angiosperm Phylogeny Group, 2016), with parsimony ancestral state reconstruction of tendrils derived from modified terminal leaflets (A) and from modified leaf tips (B).
