# Supplementary material for: Convergent Evolution and the Diverse Ontogenetic Origins of Tendrils in Angiosperms
Source: Front Plant Sci. 2018 Apr 3;9:403. doi: 10.3389/fpls.2018.00403 (PMC5891604; doi:10.3389/fpls.2018.00403)
Supplement: Supplementary file 3 [file Image3.PDF]

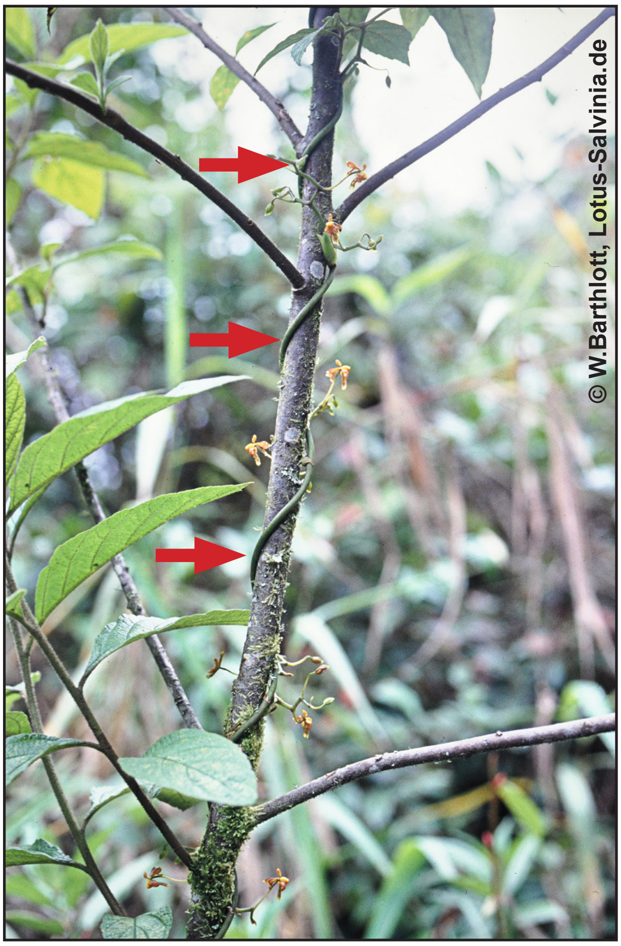

Supplementary Figure 3. Tendrils derived from the inflorescence rachis in *Oncidium* sp. (Orchidaceae, Asparagales). Arrows indicate the voluble rachis, which forms a right-handed helix.
